# Association between regulatory T cell activity and sepsis and outcome of severely burned patients: a prospective, observational study

**DOI:** 10.1186/cc8232

**Published:** 2010-01-11

**Authors:** Li-feng Huang, Yong-ming Yao, Ning Dong, Yan Yu, Li-xin He, Zhi-yong Sheng

**Affiliations:** 1 Department of Microbiology and Immunology, Burns Institute, First Hospital Affiliated to the Chinese PLA General Hospital, 51 Fu-cheng Road, Haidian District, Beijing 100048, PR China

## Abstract

**Introduction:**

To investigate the significance of changes in regulatory T cells (Tregs) activity and its relationship with sepsis, as well as outcome of patients with major burns.

**Methods:**

The periphery blood samples of 106 patients were collected on post-burn days 1, 3, 7, 14, and 21. Tregs were isolated and their phenotypes (cytotoxic T-lymphocyte-associated antigen 4 and forkhead/winged helix transcription factor p3) were analyzed by flow cytometry, and the contents of cytokines (interleukin-10 and transforming growth factor-β1) released into supernatants by Tregs were also determined by enzyme-linked immunosorbent assay kits. Gene expressions of cytokines were assessed by real-time quantitative polymerase chain reaction.

**Results:**

Expressions of Tregs phenotypes and gene/protein expression of cytokines were all elevated after burn, and there were obvious differences among patients with various burn sizes. They were also higher in septic patients than those without sepsis. Among septic patients, the expressions of Tregs phenotypes and the levels of cytokines were markedly lower in the survival group than those in patients with fatal outcome.

**Conclusions:**

Severe burn injury per se could lead to the changes in Tregs activities. Elevated levels of cytokines produced by Tregs and activation markers on Tregs surface might play an important role in the pathogenesis of sepsis and mortality in burned patients.

## Introduction

There is accumulating evidence indicating that regulatory T cells (Tregs) play important roles in the maintenance of immunologic self-tolerance and in down-regulation of various immune responses [1]. Tregs have been shown to be important in regulating the immune responses in transplant rejection, tumor immunity, infectious diseases and allergy. Thus, there has recently been an increasing interest in investigating the biology of Tregs as well as its potential application in the treatment of immunity relevant illnesses.

Many types of Treg subsets have been reported in a variety of morbid conditions. It is now clear that immune regulatory cells consist of many distinct T cell subsets [2]. Among them, CD4^+ ^Tregs have been demonstrated in a wide range of animal models and in humans [3,4], and the forkhead/winged helix transcription factor p3 (FOXP3) has been suggested to represent a reliable intracellular marker for naturally occurring Tregs [5]. Most studies on CD4^+ ^Tregs use a combination of CD25, cytotoxic T-lymphocyte-associated antigen 4 (CTLA-4), FOXP3, IL-10 and/or transforming growth factor-β1 (TGF-β1) to define Treg populations [6].

The stress response to burn injury is similar to that of severe trauma or critical illness but differs in its severity and duration. The inflammatory response is triggered immediately after thermal injury and persists for almost five weeks postburn [7]. Superimposed severe infections can result in the suppression of one or more functions of the host immune system after major burns. Multiple mechanisms have been proposed to explain infection-induced immunosuppression, including an imbalance in the cellular T helper cell (Th1/Th2) or cytokine profile, induction of anergy, depletion of effector cells and most recently the activation of CD4^+^CD25^+ ^Tregs [8]. The role of both naturally occurring CD4^+^CD25^+ ^Tregs and IL-10-secreting Tregs in infection has been the subject of several recent excellent reviews [9,10]. However, it seems that its response to trauma, burns, hemorrhagic shock, and microbial infection is associated with only a transient proinflammatory period followed by a more prolonged period of immune suppression [11]. Thus, it is speculated that there are some other factors involved in this process.

Numerous studies show that an increased burn size leads to higher mortality of burned patients [12,13]. It was also implicated that the extent of burn size might be associated with the development of sepsis. It is now believed that along with the body's hyperinflammatory response designated to eliminate the invading pathogen, mechanisms primarily aimed at controlling this initial response are initiated, but may turn out to be deleterious with immune dysfunctions and even death. A similar state of immune suppression has been described after numerous forms of severe trauma [14-16].

Although more and more evidence for immune dysfunction after sepsis has been accumulated the mechanisms underlying its development and how it acts to worsen the morbid state of the critically ill patient are yet to be elucidated. In this context, although the majority of clinical and basic researches conducted so far have focused on the roles of myeloid cell populations [17], the contribution of T lymphocytes [18,19] and, in particular, of Tregs has been somewhat ignored. Whether CD4^+^CD25^+ ^Tregs participate directly in sepsis-induced immunoparalysis resulting in poor outcomes remains to be investigated. The purpose of the present study was to investigate the significance of changes in activity of Tregs in severely burned patients, and its relation with pathogenesis of sepsis as well as the outcome of the patients following major burns.

## Materials and methods

### Participants and demography

One hundred and six patients who were admitted to our burns unit with total burn surface area (TBSA) larger than 30% were included in the present study over a time period of 10 months. Patients were resuscitated according to the Parkland formula using colloid and Ringer's lactate. Within 48 hours of admission all patients had undergone most burn wound excision for full-thickness burns, and the excision wounds were covered with available autologous skin, and allograft was used to cover any remaining open wounds. Five to ten days after healing of the donor area, the remaining wounds were totally covered with autograft skin.

The thermally injured patients were stratified into three groups according to burn size: 30 to 49% TBSA burns (group I, n = 41), 50 to 69% TBSA burns (group II, n = 34), and more than 70% TBSA burns (group III, n = 31). According to whether there was development of sepsis or not, patients were divided into sepsis group (n = 59) and non-sepsis group based on the criteria for definition of severe sepsis [20] (n = 47); then the patients with sepsis were further divided into non-survival group (n = 17) and survival group (n = 42). Twenty-five healthy volunteers served as normal controls (17 men and 8 women, mean age 28.6 ± 6.2 years, range 21 to 45 years). The periphery blood samples were collected on postburn days (PBD) 1, 3, 7, 14, and 21. The study was reviewed and approved by the Institutional Review Board of the Burns Institute, First Hospital Affiliated to the Chinese PLA General Hospital, Beijing, China. Prior to the study, each patient or the patient's legal guardian signed a written informed consent form.

### Reagents and kits

RPMI 1640, FCS, glutamine, penicillin, streptomycin, and HEPES were purchased from TianRunShanda Biotech Co. Ltd (Beijing, China). Human CD4^+^CD25^+ ^Tregs isolation kit and human fluorescein isothiocyanate (FITC)-conjugated anti-human CD4 (IgG1, Clone M-T466) were purchased from Miltenyi Biotec GmbH (Bergisch Gladbach, Germany). Antibodies used for flow cytometry analysis, including FITC-conjugated anti-human CD152 (CTLA-4, IgG2a, Clone 14D3), FITC-conjugated anti-human FOXP3 (IgG2a, κ. Clone PCH101) were purchased from eBioscience (San Diego, CA, USA). Total RNA isolation system and RT-PCR system were purchased from Promega (Madison, WI, USA). SYBR Green PCR Master MIX was purchased from Applied Biosystems (Foster City, CA, USA). ELISA kits of human IL-10 and TGF-β1 were purchased from Biosource (Worcester, MA, USA).

### Isolation of circulating Tregs

In an EDTA test tube, 10 ml of venous blood was collected. It was then diluted in Hanks' balanced salt solution, and Ficoll-Hypaque (Sigma Chemical Co., St. Louis, MO, USA) was used for isolation and preparation of peripheral blood lymphocytes. After centrifugation, the sedimentary cells were collected. The cells were isolated using MicroBeads and a MiniMACS™ separator according to the manufacturer's instructions. CD4^+ ^T cells were enriched by depletion of cells expressing CD8, CD14, CD16, CD19, CD36, CD56, CD123, TCRγ/δ and CD235a from lymphocytes with a CD4^+^CD25^+ ^Regulatory T Cell Isolation Kit. CD4^+^CD25^+ ^Tregs and CD4^+^CD25^- ^T cells were further selected according to the expression of CD25. The purity of isolated Tregs and CD4^+^CD25^- ^T cells were verified by flow cytometric analysis with anti-CD4 and anti-CD25 staining. Tregs were then cultured in RPMI 1640 FCS (10%) overnight for recovery. The supernatants were collected for the determination of IL-10 and TGF-β1 levels.

### Flow cytometric analysis

To observe the CTLA-4 expression on the surface of Tregs, cells were stained with anti-human CTLA-4-FITC antibody for 30 minutes at 4°C in the dark. Concomitantly, for detection of intranuclear FOXP3, cells were reacted with 1 ml freshly prepared fixation/permeabilization working solution for two hours at 4°C. After washing cells with one times permeabilization buffer twice, cells were stained with anti-human FOXP3-FITC antibody for 30 minutes at 4°C in the dark. After washing twice, cells were analyzed by flow cytometry using a FACScan (BD Biosciences, Mountain View, CA, USA). The fluorescence intensity was represented as a mean value.

### Cytokine measurements by ELISA

IL-10 and TGF-β1 levels were determined by ELISA, strictly following the protocols provided by the manufacturer. The color reaction was terminated by adding 100 μl of ortho-phosphoric acid. Plates were read in a microplate reader (Spectra MR, Dynex, VA, USA). The standard concentration curves for both IL-10 and TGF-β1 were from 0 to 2000 pg/ml.

### SYBR green real-time RT-PCR

Total RNA was extracted from Tregs using the single-step technique of acid guanidinium thiocyanate-chloroform extraction, according to the manufacturer's instructions. The concentration of purified total RNA was determined spectrophotometrically at 260 nm. mRNA for IL-10 and TGF-β1 in Tregs and GAPDH were quantified in duplicate by SYBR Green two-step, real-time RT-PCR. After the removal of potentially contaminating DNA with DNase I, 1 μg of total RNA from each sample was used for reverse transcription with an oligo dT and a Superscript II to generate first-strand cDNA. PCR reaction mixture was prepared using SYBR Green PCR Master Mix. Thermal cycling conditions were 10 minutes at 95°C followed by 40 cycles of 95°C for 15 seconds and 60°C for one minute on a Sequence Detection System (Applied Biosystems, Foster City, CA, USA). Each gene expression was normalized with GAPDH mRNA content. Sequences of human primer for SYBR Green PCR were shown follows: IL-10 (79 bp) - AAGGCGCATGTGAACTCCC (sense), ACGGCCTTGCTCTTG TTTTC (antisense) [21]; TGF-β1 (85 bp) - TGAACCGGCCTTTCCTGCTTCTCATG (sense), GCGGAAGTCAATGTACAGCTGCCGC (antisense) [22]; GAPDH (147 bp) -ACTTCAACAGCGACACCCACT (sense), GCCAAATTCGTTGTCATACCAG (antisense) [23].

### Statistical analysis

Data were expressed as mean ± standard deviation (SD) and analyzed with analysis of variance (ANOVA; a mixed-model, factorial ANOVA). Turkey Test was used to evaluate significant differences between groups. A *P *value of 0.05 or less was considered to indicate statistical significance.

## Results

### Demographics

One hundred and six patients with burn injury were included in the present study. The patients' demographics are illustrated in Table 1. The test for homogeneity of variance was considered and the ANOVA assumption was met. The omnibus ANOVA was also found to be significant. There was no significant difference in age among the patients with different burn size. However, there were significant differences in burn area between Group II and Group I (*P *< 0.01). The sepsis group had markedly large burn areas compared with the non-sepsis group (*P *< 0.01). Similarly, burn area in the non-survivors was much larger than that in the survivors (*P *< 0.01).

**Table 1:** Patient demographics

Variable	n	Age (years)	Burn area (TBSA%)	Average area (TBSA%)	III° area (TBSA%)
Total	106	33.6 ± 4.3	30-99	57.4 ± 5.6	33.3 ± 6.5
Group I	41	33.1 ± 6.9	30-49	39.4 ± 7.4	17.4 ± 3.9
Group II	34	29.3 ± 5.1	50-69	57.7 ± 10.7**	32.9 ± 6.3**
Group III	31	32.4 ± 6.3	70-99	82.6 ± 15.1^&&^	63.7 ± 14.5^&&^
Sepsis	59	34.3 ± 5.5	30-99	67.2 ± 11.3^##^	45.3 ± 8.6^##^
Non-sepsis	47	32.8 ± 5.7	30-72	45.0 ± 8.1	18.2 ± 3.8
Survivors	42	35.7 ± 6.8	30-88	56.2 ± 10.2^††^	29.2 ± 5.8^††^
Non-survivors	17	31.2 ± 10.5	30-99	77.1 ± 23.4	55.2 ± 16.2

Data presented as the mean ± standard deviation or percentage. TBSA = total body surface area (range); III° area = third-degree burn area. Significant difference between Group II and Group I (**P < 0.01); significant difference between Group II and Group III (^&&^*P *< 0.01); significant difference between sepsis group and non-sepsis group (^##^*P *< 0.01); Significant difference between survivors group and non-survivors (^††^*P *< 0.01).

### Isolation of CD4^+^CD25^+ ^Tregs

**Figure 1:**
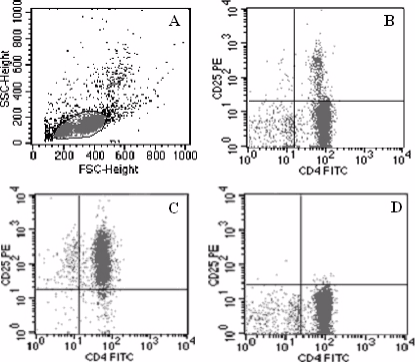
**Isolation of CD4^+^CD25^+ ^Tregs from the peripheral blood lymphocytes**. CD4^+^CD25^+^regulatory T cells (Tregs) were isolated from the peripheral blood lymphocytes in two steps by magnetic cell sorting (MACS) system according to manufacturer's instructions. **(a) **T lymphocytes before isolation and **(b) **CD4^+^T cells are shown. **(c) **The purity of positively sorted CD4^+^CD25^+ ^Tregs was 93.5 ± 1.7% with the survival rate of 96.2 ± 2.9%. **(d) **The purity of negatively sorted CD4^+^CD25-T cells was 89.6 ± 2.5%.

CD4^+^CD25^+ ^Tregs were isolated from the peripheral blood lymphocytes in two steps by a magnetic cell sorting (MACS) system. As shown in Figure 1, the purity of positively sorted CD4^+^CD25^+^Tregs was 93.5 ± 1.7% with the survival rate of 96.2 ± 2.9%. The purity of negatively sorted CD4^+^CD25^- ^T cells was 89.6 ± 2.5%.

### The phenotypic changes in Tregs after burns

**Figure 2:**
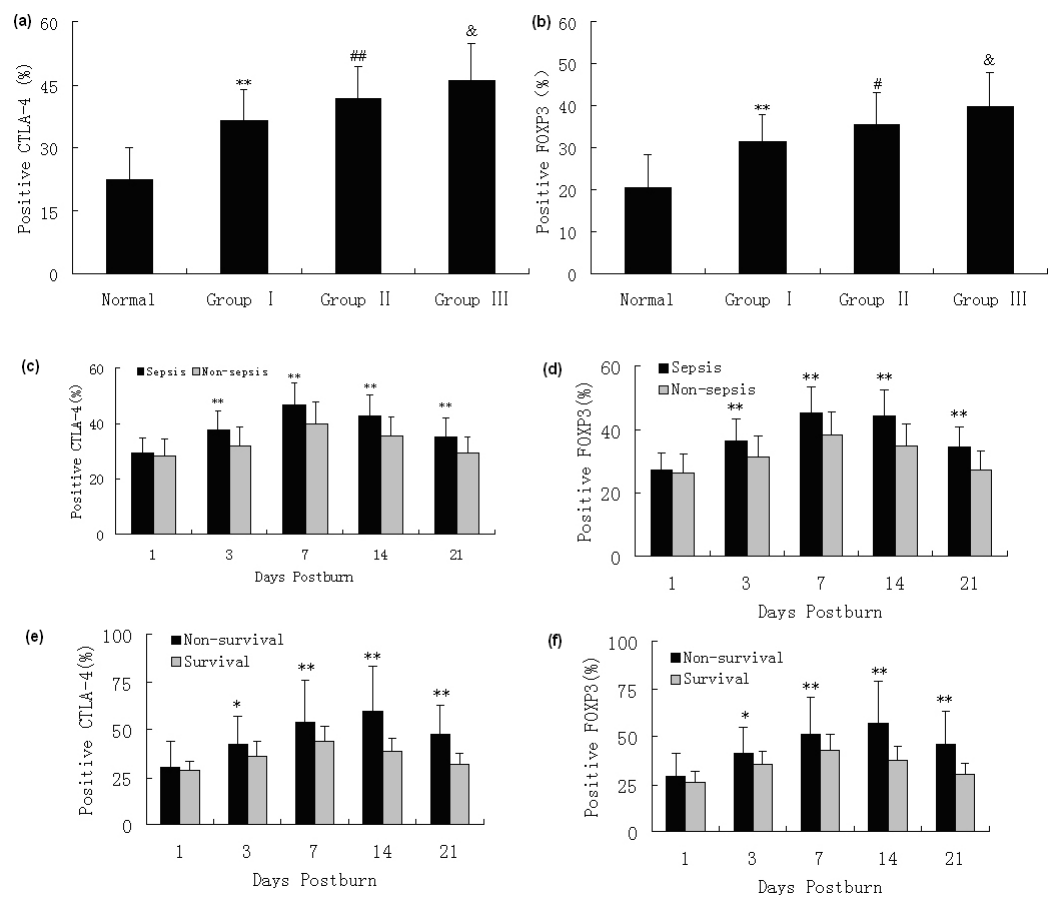
**Flow cytometric analysis of phenotypes of Tregs**. Increased expressions of CTLA-4 and FOXP3 on the surface of regulatory T cells (Tregs) from burned patients were found on postburn days (PBD) 1 to 21 compared with normal controls, and there were obvious differences among patients with various burn sizes (**(a and b) **a mean of all days). The expressions of CTLA-4 and FOXP3 were significantly higher in patients with serious burns during the whole observational period, and **(c and d) **they were much higher in septic patients than those without sepsis on PBD 3 to 21. **(e and f) **Among septic patients, the expressions of CTLA-4 and FOXP3 in the survival group were obviously lower than those in non-survival group on PBD 3 to 21. * *P *< 0.05, ** *P *< 0.01, Group I vs. normal group or sepsis group vs. non-sepsis group or non-survivors vs. survivors; #*P *< 0.05, ## *P *< 0.01, Group II vs. Group I; &*P *< 0.05, Group III vs. Group II.

To investigate the changes in Treg phenotypes, these cells were analyzed at different time points and in different groups after burns. A three (Group) times five (Day) mixed-model, factorial ANOVA was conducted. As shown in Figure 2, increased expressions of CTLA-4 and FOXP3 were found to be enhanced on the surface of Tregs from burned patients on PBD 1 to 21 compared with normal controls, and there were obvious differences among patients with various burn sizes (*P *< 0.05 or *P *< 0.01). The expressions of CTLA-4 and FOXP3 were significantly higher in patients with serious burns at all time points, and they were even higher in septic patients than those without sepsis on PBD 3 to 21 (*P *< 0.01). Among septic patients, the expressions of CTLA-4 and FOXP3 in the survival group were significantly lower than those with fatal outcome on PBD 3 to 21 (*P *< 0.05 or *P *< 0.01).

### Changes in protein and gene levels of cytokines released by Tregs

**Figure 3:**
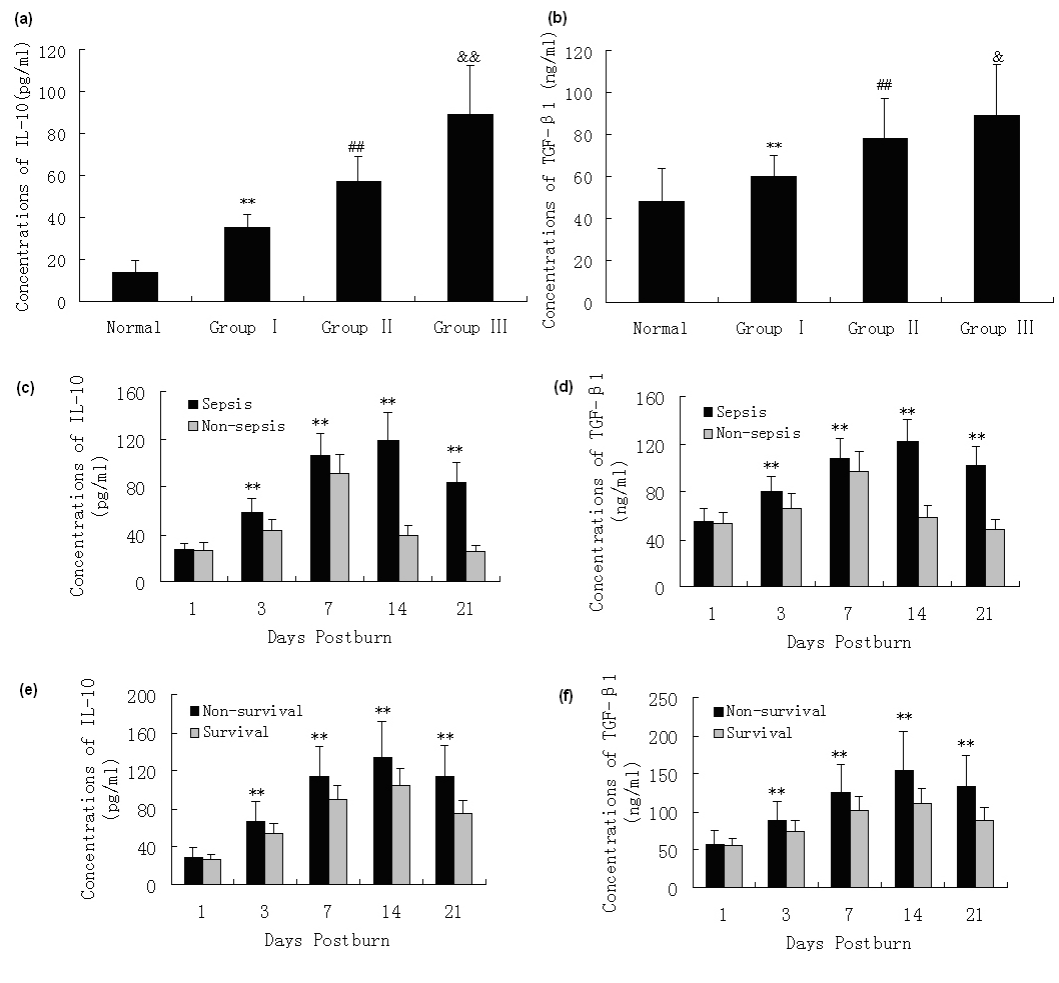
**ELISA analysis of IL-10 and TGF-β1 levels in Tregs supernatants**. Elevated protein expressions of IL-10 and TGF-β1 in regulatory T cells (Tregs) from burned patients were detected on postburn days (PBD) 1 to 21 in comparison to normal controls, and there were obvious differences among patients with different extent of burn injury (**(a and b) **a mean of all days). Protein levels of **(c) **IL-10 and **(d) **TGF-β1 in Tregs were significantly higher in septic patients than those without sepsis on PBD 3 to 21. Among septic patients, **(e) **IL-10 and **(f) **TGF-β1 levels in the survivors were obviously lower than those with non-survivors on PBD 3 to 21. **P < 0.01, Group I vs. normal group, or sepsis group vs. non-sepsis group or non-survivors group vs. survivors group; ^##^*P *< 0.01, Group II vs. Group I; ^&^*P *< 0.05, ^&&^*P *< 0.01, Group III vs. Group II.

**Figure 4:**
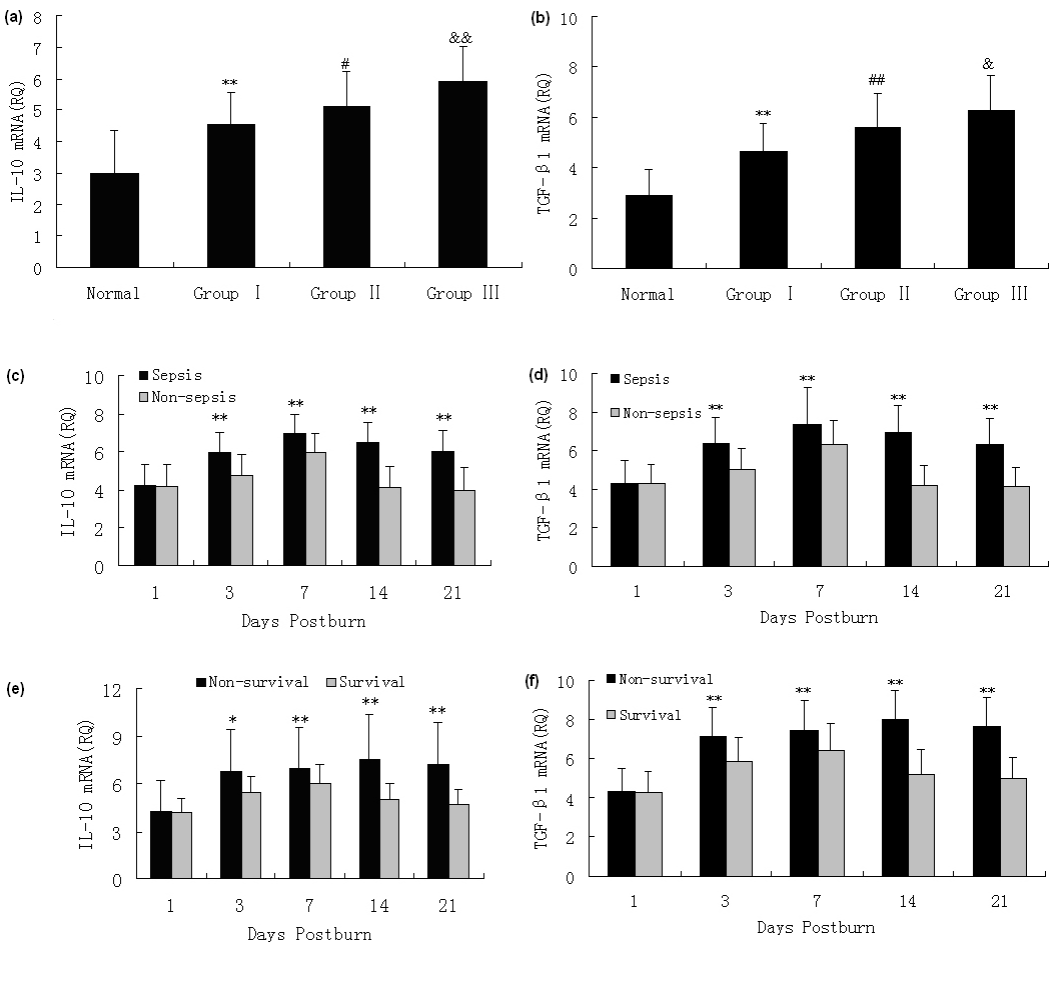
**SYBR green real-time RT-PCR analysis for mRNA expression of IL-10 and TGF-β1 in Tregs**. Enhanced gene expressions of **(a) **IL-10 and **(b) **TGF-β1 in regulatory T cells (Tregs) from burned patients were detected on postburn days (PBD) 1 to 21 in comparison with normal controls, and there were obvious differences among patients with different extent of burn injury. mRNA expressions of **(c) **IL-10 and **(d) **TGF-β1 in Tregs were significantly higher in septic patients than those without sepsis on PBD 3 to 21. Among septic patients, **(e) **IL-10 and **(f) **TGF-β1 mRNA expressions in the survival group were markedly lower than those with fatal outcome on PBD 3 to 21. *P < 0.01, **P < 0.01, Group I vs. normal group or sepsis group vs. non-sepsis group, or non-survivors group vs. survivors group; ^#^*P *< 0.01, ^##^*P *< 0.01, Group II vs. Group I; ^&^*P *< 0.05, ^&&^*P *< 0.01, Group III vs. Group II.

The capacity of Tregs to produce IL-10 and TGF-β1, which are two of the markers of function of Tregs, was analyzed in the present experiment. As shown in Figures 3 and 4, elevated protein and gene expressions of IL-10 and TGF-β1 in Tregs from burned patients were detected on PBD 1 to 21 in comparison with normal controls, and there were marked differences among patients with different extents of burn injury (*P *< 0.05 or *P *< 0.01). The protein and gene expressions of IL-10 and TGF-β1 in Tregs were significantly higher in septic patients than those without sepsis on PBD 3 to 21 (*P *< 0.05 or *P *< 0.01). Among septic patients, the expression levels of IL-10 and TGF-β1 in the survival group were obviously lower than those with non-survival group on PBD 3 to 21 (*P *< 0.05 or *P *< 0.01).

## Discussion

Severe burn injury induces detrimental changes in immune function, often leaving the host highly susceptible to developing life-threatening opportunistic infections. Advances in our understanding of how burn influences host immune response suggest that thermal injury causes a phenotypic imbalance in the regulation of Th1- and Th2-type immune responses [24]. The immune response to infection represents a complex balance between the successful induction of proinflammatory antipathogen response and anti-inflammatory response required to limit damage to host tissues. The vast majority of clinical and basic science research on the immune consequences of burn injury and sepsis conducted during the past three decades has focused mainly on the roles of macrophages, neutrophils, and, to a lesser extent, conventional T lymphocytes [25,26]. During recent years, however, it has become increasingly clear that minor subsets of innate immune cells, innate regulatory lymphocytes in particular, are central to processes involved in both protective immunity and immunopathology [27]. Tregs undoubtedly play an important role in controlling this balance during infection, and the results can range from highly detrimental to the host to highly beneficial to both the host and pathogen [28].

In our previous observations, significant proliferation of splenic T cells and IL-2 as well as IL-2Rα expression on T cells were simultaneously suppressed to a certain extent on PBD 1 to 7 in rats [29]. Nuclear factor of activated T cell activity of splenic T cells was markedly down-regulated on PBD 1 to 3. It was revealed that T cells were polarized to Th2 cells after burn injury. These data indicate that there is a marked suppression of T cell function following major burns. To collaborate with other findings, it has been reported that Tregs in mice can inhibit the proliferation of T cell and release of cytokine for polarization to antigen-specific Th1 cells after acute insults [30]. Similarly, we recently reported increased Treg activity after thermal injury in rats [31]. Because severe burn injury triggers both excessive inflammation and suppressed adaptive immunity, we would expect that there might be an activation of immune activity of Tregs isolated from burn-injury patients. Therefore, a major objective of this study was to define how thermal injury influenced the maturation of Tregs in peripheral blood in severely burned patients. We considered that this is an important question to be addressed as regulation of Th1- and Th2-type responses against infectious pathogens by Tregs can markedly affect host survival [30].

In the current study, increased expressions of CTLA-4 and FOXP3 on the surface of Tregs from burned patients were observed on PBD 1 to 21 compared with normal controls. This finding was consistent with a previous report that was published prior to the identification of Tregs [32]. In that study, the authors demonstrated that cell-surface CD25 expression on CD4^+ ^T cells increased by five days after injury and remained elevated for at least two weeks. Meantime, it was presumed the increase in CD25 expression was due to injury-induced CD4^+ ^T cell activation. However, we demonstrate here that the increased percentage of circulating Tregs in our burned patients was attributable to both T cell activation and a significant increase in the percentage of CD4^+^CD25^high ^cells. This increase in circulating CD4^+^CD25^high ^T cells may represent Tregs expansion or possibly migration of these cells from immunologically active sites instigated by the injury. These are important mechanistic issues that can be studied in more detail using animal models of injury.

Numerous studies have shown that an increased burn size leads to increased mortality in burn patients [12,33]. In a large prospective clinical trial, Jeschke has indicated that different burn sizes are associated with differences in intensity of inflammation, in body composition, in protein synthesis, and in organ function. In the present study *in vivo*, we found there were obvious differences in the expressions of CTLA-4 and FOXP3 on the surface of Tregs among patients with various burn sizes. Taken together, these data suggest that acute insults can induce or amplify CD4^+^CD25^+ ^Tregs function and that CD4^+^CD25^+ ^T cells contribute to the development of postinjury immunosuppression.

Using a mouse burn injury model, Ni Choileain noticed that injury per se significantly enhances Tregs function [33]. Such increase in Tregs activity was apparent on day 7 after injury and was restricted to CD4^+^CD25^+ ^T cells in lymph nodes draining the injury site. Moreover, our recent report implicated that the injury-induced increase in Tregs activity was cell-contact dependent and was mediated in part by increased cell surface TGF-β1 expression [31]. Depending on the different settings, cytokines (including TGF-β1 and IL-10) as well as direct cell killing of conventional T cells and antigen presenting cells (APCs) by the Tregs have been proposed as the one of the mechanism of immunosuppression [34-36]. Similarly, our results in this study showed that gene/protein expression of IL-10 and TGF-β1 in Tregs from burn patients was augmented on PBD 1-21 in comparison to normal controls, and there were obvious differences among patients with different extent of burn injury. It appears that serious burn injury induces activitiy of Tregs resulting in high expressions of certain phenotypes, and this enhanced Tregs activity might play a key role in modulating cell-mediated immunity of T lymphocytes.

Sepsis and subsequent multiple organ dysfunction syndrome are frequent complications of major trauma or burns, and remain to be the most common cause of morbidity as well as mortality in critical illnesses. It is well known that marked immune depression is critically involved in the development of sepsis. Immunoparalysis has recently be thought to be a possible cause of explaining the failure of numerous clinical therapeutic trials in septic shock [37]. Severe burn injury induced a marked reduction in HLA-DR expression at both protein and messenger RNA levels [38]. Its persistent decrease was associated with mortality and the development of septic complications [39]. However, whether sepsis and mortality after burns are due to inflammation, immune suppression or other pathophysiologic contributing factors is not entirely elucidated. Furthermore, it is also not very clear whether Tregs induced by severe burns can contribute to the development of sepsis and outcome of the patients.

The studies presented here described that the expressions of activation markers of Tregs and cytokines produced by Tregs were significantly higher in patients with serious burns at all time points, and they were much higher in septic patients than those without sepsis on PBD 3 to 21. Among septic patients, the expressions of these parameters in the survival group were markedly lower than those with fatal outcome on PBD 3 to 21. These findings support the concept that CD4^+^CD25^+ ^Tregs contribute to the control of immune response after being affected by thermal injury and sepsis. The persistence of a pronounced immunoparalysis induced by Tregs after severe sepsis is associated with a poor outcome after burns. Recently, similar findings have been reported by others [40-42]. Some authors suggest that although CD4^+^CD25^+ ^Tregs induced by IL-10 seem to contribute to sepsis-induced suppression of lymphoid dependent immunity, the removal of CD25^+ ^cells does not provide a survival advantage or disadvantage.

We therefore speculated that Tregs might also play an essential role in initiating effective immunosuppression response to sepsis. This may be related to their ability to interact with components of the innate and adaptive immune response, and to their potentiality to be activated nonspecifically by bacterial products and/or cytokines, and to regulate through direct cell-cell and/or soluble mediators. It is our hope that a better understanding of the mechanism through which those rare lymphocyte subsets, which is found to exert such a profound effect on the immune response, may help in improving our clinical ability not only in diagnosis but also in treatment for the critically septic individual.

## Conclusions

In summary, severe burn injury *per se *could result in activation and maturation of Tregs, thus invoking its immunodepressive activity to the full extent, finally leading to immunosuppression. Elevated levels of cytokines produced by Tregs and activation markers on Tregs surface might play an important role in the pathogenesis of sepsis and mortality in burned patients.

## Key messages

• Severe burn played an important role in activation and expansion of Treg cells. This feature might allow Treg to function for a prolonged period of time to regulate immune responses and induce suppression of T lymphocyte immune function.

• The elevated levels of cytokines producted by Treg and activation markers on Treg surface may also be involved in increased burn sizes, sepsis and mortality of burned patients.

• This suggested that Treg might have a potential for suppressing the proliferation and cytokine production of T cells *in vivo*. It also suggested that the regulation of Treg cells as a cellular therapy might be important to the Th1/Th2 cytokine balance in burned patients and sepsis patients.

## Abbreviations

ANOVA: analysis of variance; bp: base pair; CTLA-4: cytotoxic T-lymphocyte-associated antigen 4; ELISA: enzyme-linked immunosorbent assay; FCS: fetal calf serum; FITC: fluorescein isothiocyanate; FOXP3: the forkhead/winged helix transcription factor p3; IL: interleukin; MACS: magnetic cell sorting; PBD: postburn days; RT-PCR: reverse transcription polymerase chain reaction; SD: standard deviation; TBSA: total body surface area; TGF-β1: transforming growth factor-β1; Tregs: regulatory T cells.

## Competing interests

The authors declare that they have no competing interests.

## Authors' contributions

YMY supervised the entire project and wrote the manuscript with LFH and with comments from all coauthors. LFH and ZYS participated in the study design. LFH, YMY, YY, and LXH conducted the clinical study. ND processed the data analysis. All authors read and approved the final manuscript.
